# Supplementary figures and images for: Portosystemic shunting prevents hepatocellular carcinoma in non-alcoholic fatty liver disease mouse models
Source: PLoS One. 2023 Dec 29;18(12):e0296265. doi: 10.1371/journal.pone.0296265 (PMC10756526; doi:10.1371/journal.pone.0296265)

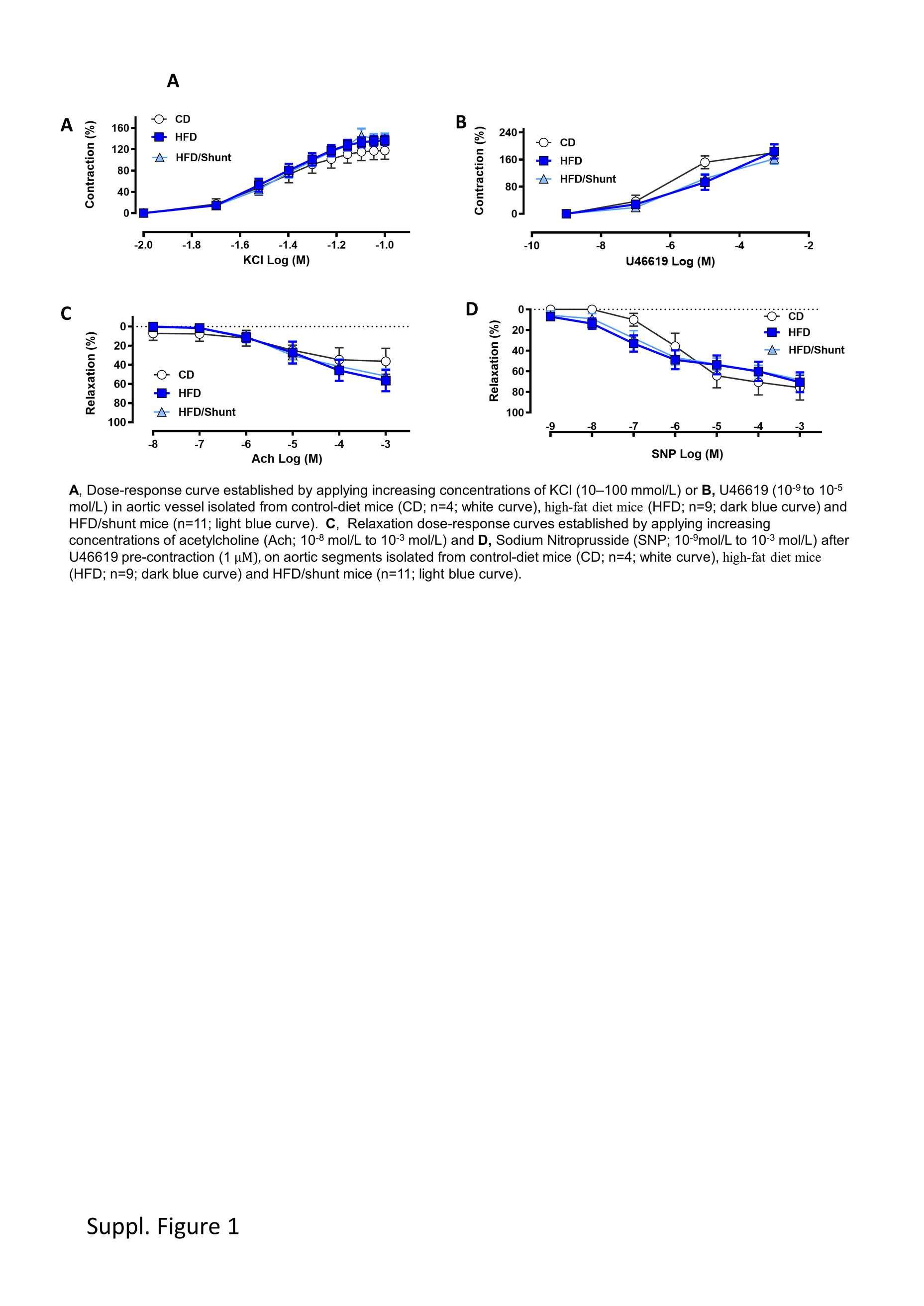

Supplement: S1 Fig — Dose-response curve established by applying increasing concentrations of KCl (10–100 mmol/L) (A) or U46619 (10–9 to 10–5 mol/L)(B) in aortic vessel isolated from control-diet mice (CD; n = 4; white curve), high-fat diet mice (HFD; n = 9; dark blue curve) and HFD/shunt mice (n = 11; light blue curve). Relaxation dose-response curves established by applying increasing concentrations of acetylcholine (Ach; 10–8 mol/L to 10–3 mol/L) (C) and Sodium Nitroprusside (SNP; 10-9mol/L to 10–3 mol/L) after U46619 pre-contraction (1 μM) (D), on aortic segments isolated from control-diet mice (CD; n = 4; white curve), high-fat diet mice (HFD; n = 9; dark blue curve) and HFD/shunt mice (n = 11; light blue curve). (TIF) [file pone.0296265.s001.tif]

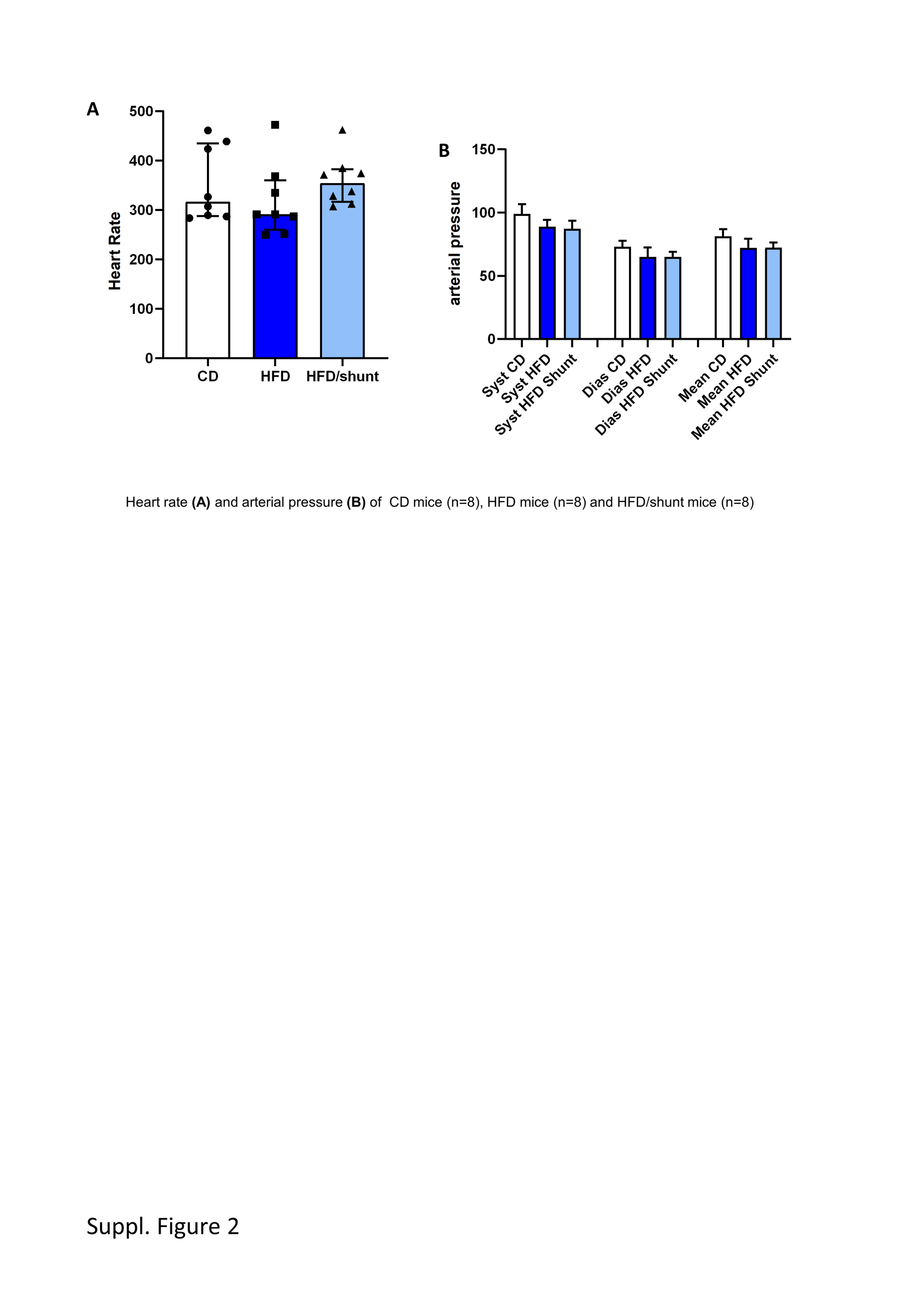

Supplement: S2 Fig — Heart rate (A) and arterial pressure (B) of CD mice (n = 8), HFD mice (n = 8) and HFD/shunt mice (n = 8). (TIF) [file pone.0296265.s002.tif]

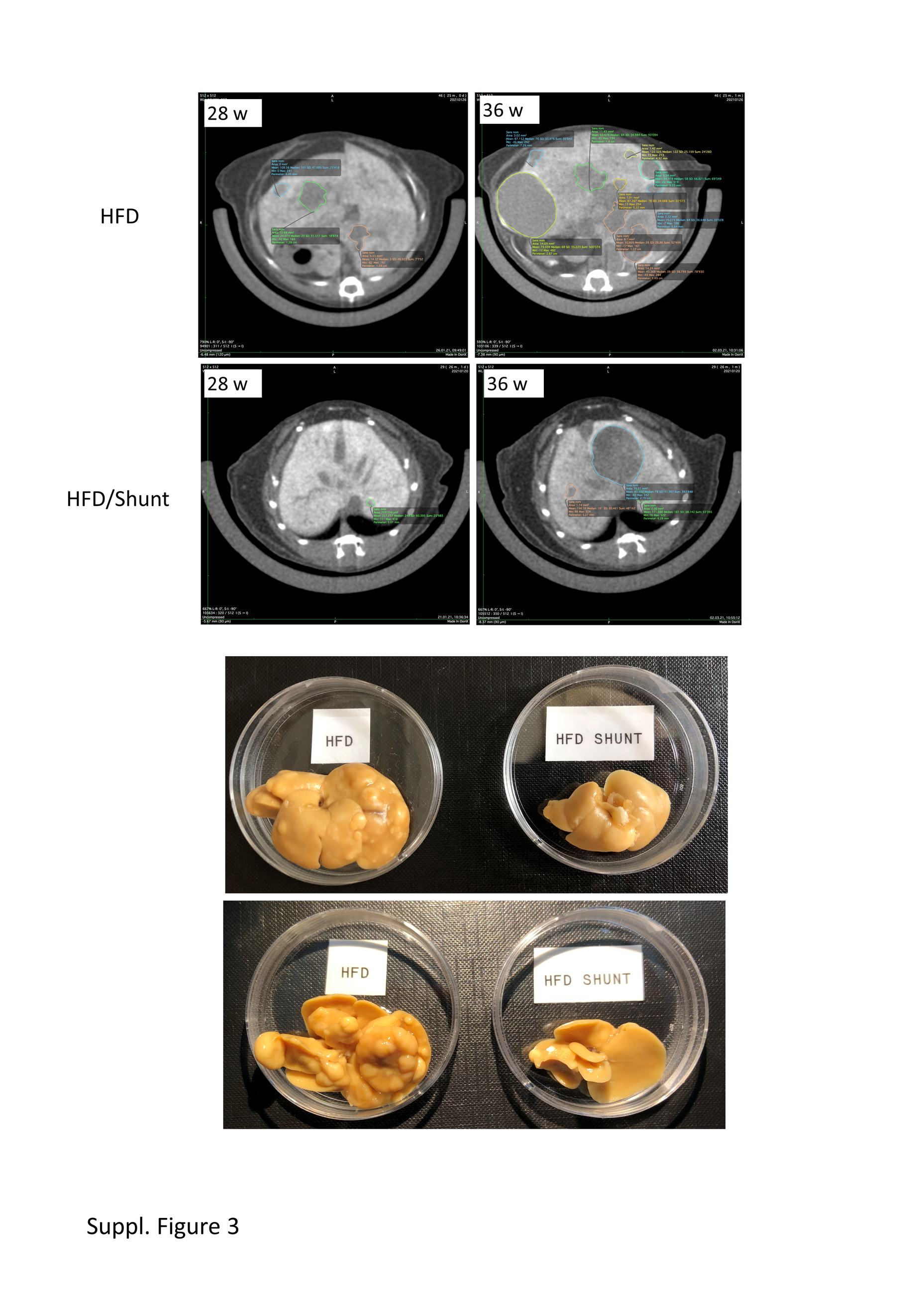

Supplement: S3 Fig — MicroCT sections of livers at 28 weeks and 36 weeks and pictures of livers at 40 weeks (HFD and HFD/Shunt). (TIF) [file pone.0296265.s003.tif]

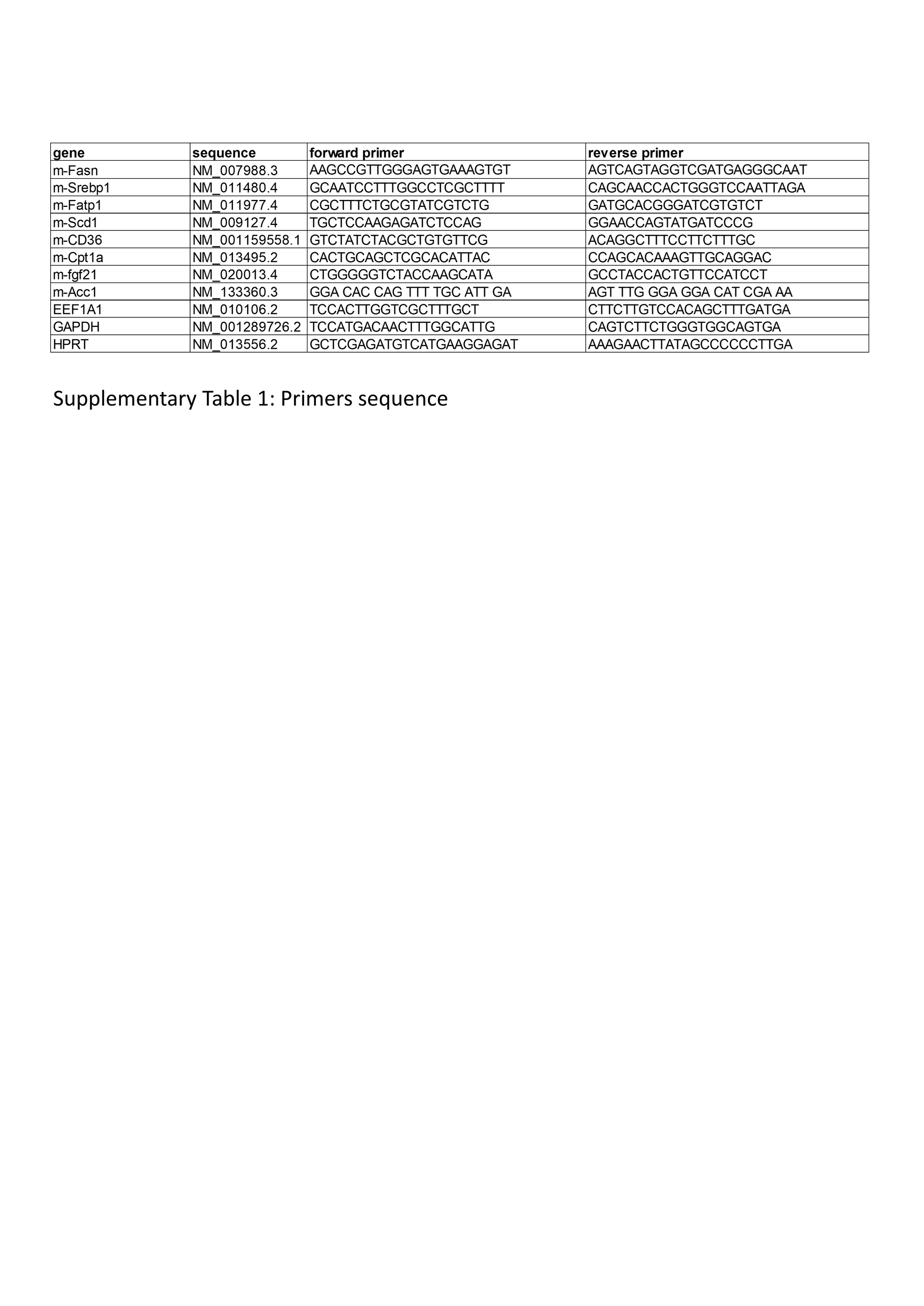

Supplement: S1 Table — (TIF) [file pone.0296265.s004.tif]
