# Supplementary material for: Portosystemic shunting prevents hepatocellular carcinoma in non-alcoholic fatty liver disease mouse models
Source: PLoS One. 2023 Dec 29;18(12):e0296265. doi: 10.1371/journal.pone.0296265 (PMC10756526; doi:10.1371/journal.pone.0296265)
Supplement: Attachment — Submitted filename: PointByPointResponse.docx [file pone.0296265.s006.docx]

Academic Editor comment:
I have received both reviewers’ comments concerning the submission to PloS ONE. Reviewer #1 made suggestions and accepted the manuscript after the R1 version. However, reviewer #2 has expressed serious concerns about the results of the manuscript. Unfortunately, we cannot accept this manuscript version due to the concerns described below. The authors should take advantage of the criticism and resubmit the article, as a new submission, with the necessary modifications.

*We would like to thank you for your positive effort in improving our study and manuscript. However, we would also ask you to reconsider your decision to reject our manuscript.*

*Although important, the points raised by Reviewer 2 do not challenge nor the design of the study, nor its results.*

Reviewer #1:

The authors have now fully addressed all of the reviewer's questionings. The link directing to the raw data is now operational and the work is suitable for publication.

*Thank you for your help to improve our manuscript.*

Reviewer #2:

1. The link is now functional, but it takes a long time to download, makes it difficult for the readers to reach the information. It is no exaggeration to say that it is not functioning, and it is a big problem. Even after the reviewer have read the revised manuscript and finished writing the comments, it was not possible to download even a single file.

*Our raw data are hosted on the official repository of the University of Geneva (please see* [*https://yareta.unige.ch/home*](https://yareta.unige.ch/home)*), which respects the FAIR principles (FAIR Principles: Findable, Accessible, Interoperable, Reusable). It is true that the uploaded files are extremely heavy as they include the images of the CT scans (76.3 GB in total). It is true also that it takes some time to download the heaviest files (8 minutes for the 45.5 GB file on our computer). However, the proposed technical option is currently the best we can offer (it is approved by the Swiss National Funding agencies). We understand it takes some time to obtain the files, but we also feel that it should not be seen as rejection criterion*

2. The CT images are the same as in the previous version, and the liver cross-sectional area is different between HFD and HFD/shunt, indicating that the observed area of the liver is different.
In addition, in the HFD CT image, the right and left halves of the perimeter are not continuous, and an unnaturally straight liver tissue is depicted in the middle part, a finding that cannot be observed in vivo.

*Regarding the proposed CT images: We kindly suggested keeping the proposed images as they show the same tumors over the two assessed time points. Keeping the same level on the mouse (cut at the level of the same vertebra for example) would lead to picturing different HCCs because of the deformation of the liver linked to HCC growth. Again, we would be happy changing this if Reviewer 2 wishes it. In line with the next question, we now also include illustrative movies of the presented livers with and without shunt.*

*In the HFD CT image, “the right and left halves of the perimeter are not continuous” because they show two different liver lobes (by chance just showing on the midline). Reviewer 2 also states that “an unnaturally straight liver tissue is depicted in the middle part, a finding that cannot be observed in vivo”. To confirm that the images are a true representation of the reality, please see the attached movie (HFD.wmv) which better pictures this point. We have also included the movies of two mice with and without shunt as a supplementary material.*

3. The observation direction of the excised liver tissue was also completely opposite in the two groups. Photographs of the excised organs are usually taken from the same direction. In addition, a large tumor mass was observed in the HFD group, which is similar in size to that observed in the HFD/Shunt group. The reviewer got the impression that it is very artificial.

*It is true that the proposed liver pictures were not taken from the same side of the liver. It was a way to better show the HCCs. We would be happy providing the attached ones instead demonstrating both sides of the liver (SupplFig3). Here again, we would respectfully argue that this point should not motivate a rejection.*

*Again, we work everyday in performing rigorous experiments with appropriate replicates and controls. We also understand the high standard linked to publishing in PLOS One. However, we would again ask you to reconsider your decision regarding our manuscript.*
