# Supplementary material for: Portosystemic shunting prevents hepatocellular carcinoma in non-alcoholic fatty liver disease mouse models
Source: PLoS One. 2023 Dec 29;18(12):e0296265. doi: 10.1371/journal.pone.0296265 (PMC10756526; doi:10.1371/journal.pone.0296265)
Supplement: Attachment — Submitted filename: PointByPointResponse.docx [file pone.0296265.s007.docx]

Reviewer #1:

Reviewer #1: The authors presented the effect of portosystemic shunting on the prevention of HCC development in NAFLD mouse models. The phenomena, reduction of portal pressure and decreased incidence of HCC development are clearly demonstrated; however, the direct association of the modification of gut-liver axis by the shunting on HCC prevention is still unclear. Therefore, authors need to demonstrate the modification of gut-liver axis by showing for e.g. microbiota changes and the association of the shunting on bile acid and/or oxygenation.

*Thank you for this comment. Unfortunately, we do not have access to feces samples allowing us to perform microbiota analysis. In an effort to investigate the role of portal hypertension and it reduction by shunt, we analyzed histological alterations of the proximal bowel. HFD-fed animals developed an increased villi length, a decreased percentage of goblet cells and an increased number of lymphatic vessels compared to normal diet animals. The shunt restores the number of lymphatic vessels, suggesting a decreased inflammation in the bowel and a normalization of the lipids absorption. These results were added in the Figure 5.*

*In addition, we performed PCR in order to explore whether the alteration we demonstrate in bile acids were correlated to changes in the expression of bile acids transporters or receptors in the proximal ileum. However, we found no differences in their expression between HFD-fed mice and HFD-fed mice with shunt. This suggests the changes we observed were not linked to a decreased absorption of conjugated bile acids in the intestine.*

Expression level (2-ddCt) of some bile acid transporters and bile acids receptors in the proximal ileum (n=6/group). P value (right bottom) compared to HFD no shunts group.

*In conclusion, we cannot argue in favor of changes in the gut microbiome after shunting. However, we were able to demonstrate shunting normalized lymphangiogenesis and conjugated biliary acids levels, suggesting a restored intestinal function.*

Expression level (2-ddCt) of some bile acid transporters and bile acids receptors in the proximal ileum (n=6/group). P value (right bottom) compared to HFD no shunts group.
